# Agro-Industrial Protein Waste and Co-Products Valorization for the Development of Bioplastics: Thermoprocessing and Characterization of Feather Keratin/Gliadin Blends

**DOI:** 10.3390/molecules28217350

**Published:** 2023-10-30

**Authors:** Carol López-de-Dicastillo, Joaquín Gómez-Estaca, Gracia López-Carballo, Rafael Gavara, Pilar Hernández-Muñoz

**Affiliations:** 1 Packaging Group, Institute of Agrochemistry and Food Technology (IATA-CSIC), Av. Agustín Escardino, 7, 46980 Paterna, Spain; glopez@iata.csic.es (G.L.-C.); rgavara@iata.csic.es (R.G.); 2 Institute of Food Science, Technology and Nutrition (ICTAN-CSIC), Calle José Antonio Novais 10, 28040 Madrid, Spain; jgomez@ictan.csic.es

**Keywords:** feather keratin, wheat gliadins, packaging, biopolymer, valorization

## Abstract

Biopolymers based on plant and animal proteins are interesting alternatives in the development of films with future prospects as food packaging. Considering that in recent years there has been an increasing interest in the valorization of agro-industrial residues and by-products and that the blending of polymers can lead to materials with improved properties, in this work, keratin-rich feather fibers and gliadins were blended at different ratios in order to develop sustainable and biodegradable films. Control gliadin G100, feather F100 films, and their blends at 3:1 (G75F25), 2:2 (G50F50), and 1:3 (G25F75) ratios were successfully developed through thermoprocessing. The physical properties were differentiated as a function of the concentration of both polymeric matrices. Although gliadins showed higher hydrophilicity as confirmed by their highest swelling degree, films with high gliadin ratios exhibited lower water vapor permeability values at low and medium relative humidities. On the other hand, the feather fiber-based films displayed the highest Young’s modulus values and provided an oxygen barrier to the blends, principally at the highest relative humidity. In conclusion, the blend of these protein-based polymers at different ratio resulted in interesting composites whose physical properties could be adjusted.

## 1. Introduction

The pollution of the environment caused by the accumulation of plastic waste has become one of the most pressing environmental problems requiring attention. This is mainly attributable to the increase in disposable plastic products that take a long time to degrade, and the food-packaging industry is a major contributor to this waste. In order to address the accumulation of plastic residues, the use of non-renewable resources, and the reduction of pollutants reaching the sea, European and global strategies, such as the European Green Deal, and regulations, such as the Single-Use Plastics Directive (Directive 2019/904/EU), have been established that include a number of targets related to the use and management of plastic resources to be met in the coming years [1,2,3]. For example, the European Plastics Strategy plans that, by 2030, all packaging should be reusable, recyclable, or compostable. Thus, nowadays, the search and interest of the food-packaging sector in new compostable polymers produced from renewable resources and, particularly, from the revaluation of agro-industrial residues has grown considerably. In this regard, protein-based biopolymers have become a notable research topic thanks to their biodegradability, large availability, and good barrier to gases [4]. Although the use of protein-based films has been extensively researched, they commonly present low stability under humid conditions and low flexibility and elongation at break values. These characteristics are of great importance in the performance as food packaging, and therefore, the search for strategies for improving their overall technological properties has driven their chemical modification through the addition of crosslinkers, plasticizers, or other plastic additives and/or the blending of polymers [5,6,7].

Gliadins, wheat protein by-products of the starch industry, consist of a heterogeneous group of single polypeptide chains associated via hydrogen bonding and hydrophobic interactions [8,9]. Gliadins are an interesting biopolymer characterized by good viscoelasticity, thermo-plasticity, and film-forming properties [9,10], but some of their characteristics, such as poor mechanical properties and weak water stability, need to be improved in order to extend their use into food packaging applications.

Keratin is a fibrous structural protein that is a key component of the outer layer of human skin, hair, nails, feathers, horns, and the epidermal tissues of animals. Keratin has been widely used in biomedical applications, such as drug delivery vehicles, bone tissue engineering, and wound healing, as well as the production of biodiesel/biogas, animal feed, and bioplastics [7,11,12]. Keratin-derived materials have shown excellent attributes, such as biocompatibility, biodegradability, and mechanical durability [13,14]. The cost of keratin extraction can vary widely depending on several factors, including the source of keratin, the extraction method used, the scale of production, and the quality of the final product; nevertheless, it is usually a tedious and expensive process [15,16]. In addition, the recovery of biopolymers from agro-industrial residues has recently emerged as an innovative method of finding additional renewable sources. In this sense, chicken feathers represent a valuable residue from the poultry processing industry that is commonly discarded. Feathers contain approximately 91% keratin, 1% fat, and 8% water [6,17,18], and therefore, they can be a great source of keratin proteins and biopolymer production. Some works have already used practically raw feathers directly in the fabrication of novel materials, minimizing the treatment of waste from chicken production and promoting the circular economy. Besides this, the use of solvents, water, electricity, and, in consequence, carbon footprint could be reduced. A previous work blended chicken feathers with polylactic acid (PLA) biopolymer at 5 and 50% of feathers respect to the total material weight [7], and the effect of the addition of feathers on the biodegradation of the resulting composites was studied. Shanmugasundaram et al. (2018) demonstrated the potential application of fabricated chicken feather keratin/polysaccharides blends for nonwoven wound dressing biomaterials [6].

In this work, feather and gliadin blends have been developed and characterized in order to analyze their suitability as sustainable food packaging materials. The novelty of the present work lies both in the higher incorporation of chicken feather fiber residues into a final composite and the characterization of the physical and morphological properties of the resulting blends. The compatibility and the performance of blends for packaging purposes were studied through the analysis of their optical, morphological, thermal, mechanical, and barrier properties.

## 2. Results

### 2.1. Morphological and Structural Properties

**Figure 1:**
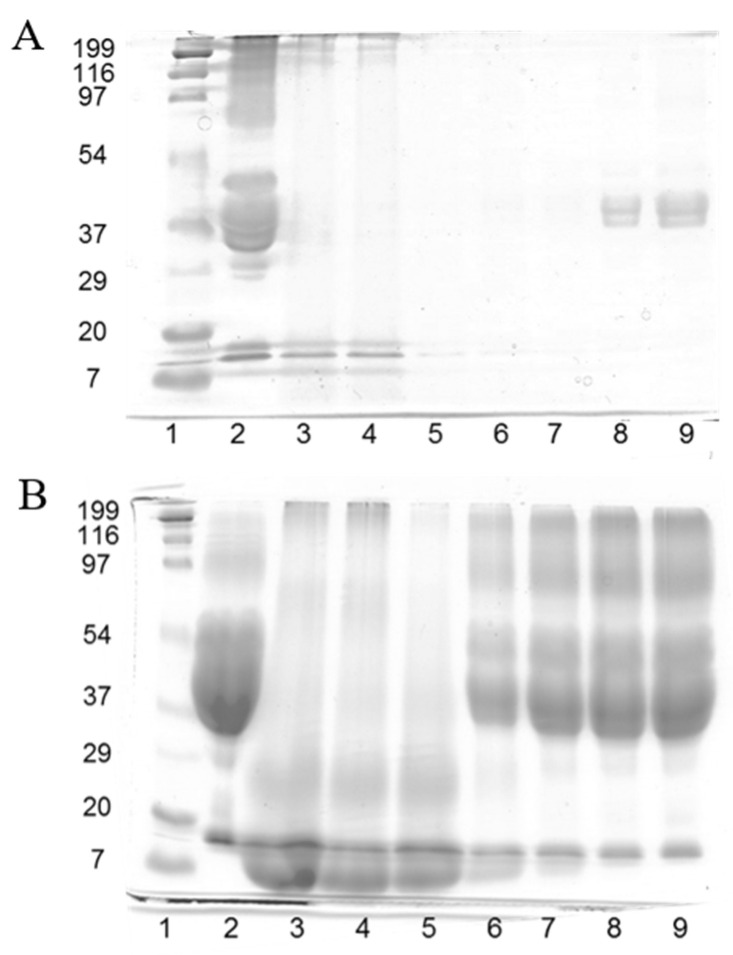
SDS-PAGE patterns in the absence of 2-mercaptoethanol (**A**) and the presence of 2-mercaptoethanol (**B**). Samples are: (1) molecular weight marker; (2) gliadin resin; (3) plain feathers; (4) reduced feathers; (5) F100 film; (6) G25F75 film; (7) G50F50 film; (8) G75F25 film; and (9) G100 film.

Figure 1 shows the SDS-PAGE patterns of gliadin resin and plain and reduced feathers and the corresponding thermopressed developed films. The pattern of the gliadin resin that was not subjected to reducing conditions with 2-ME (Figure 1A, lane 2) displayed typical poorly defined protein bands corresponding to gliadins in the 37–50 kDa range [9]. On the other hand, a considerable amount of high-molecular-weight protein was observed as a diffuse band in the 95–200 kDa range, contrary to the results shown by native gliadins. Due to the fact that this diffuse protein band of high molecular weight mostly disappeared under reducing conditions (Figure 1B, lane 2), it is likely to be composed of gliadin units cross-linked via SH-SS interchange reactions formed during the drying of the gliadin solution to obtain the resin.

Feather keratins are composed of approximately 20 proteins that differ by only a few amino acids, and they have similar molecular weights of 10.4 kDa [19,20]. Plain feathers (Figure 1A, lane 3) scarcely manifested protein bands, owing to the high degree of cross-linking and insolubility of native feather keratin fibers [20]. In fact, a considerable amount of undissolved feathers was clearly visible in the denaturation buffer. Similar results were obtained with feathers that were subjected to the reducing treatment with Na_2_SO_3_ (Figure 1A, lane 4). F100 film (Figure 1A, lane 5) did not exhibit any protein band, being indicative of the reorganization of the reduced protein fragments observed in lane 4 during film thermopressing to form a polymeric matrix that was insoluble under non-reducing denaturation conditions. The same events occurred with the G25F75 (Figure 1A, lane 6) and G50F50 (lane 7) films. G75F25 and G100 protein films (Figure 1A, lanes 8 and 9, respectively) presented a short amount of protein in the lanes, indicating the high insolubility of the films under unreduced conditions, and their protein bands were in the region of gliadins.

When analyzing the SDS-PAGE patterns under reducing conditions (Figure 1B), a high amount of protein in the 10 kDa region in lanes 3 and 4 was observed, indicating that feather fibers were reduced to render keratin. The F100 film (lane 5) displayed a similar pattern to that of plain and reduced feathers, indicating that the film protein network was mainly stabilized by disulphide bonds. The G100 film (lane 9) exhibited a diffuse protein band of high density in the 35–200 kDa range, derived from the reduction of S-S bonds that stabilized the structure of gliadin film. Blended films showed characteristics of the films elaborated with the single components, including protein fragments of high (35–200 kDa) and low (10 kDa) molecular weights, in relative proportions correlated to the amount of each polymer in the blend. These results indicated the high implication of disulphide bonds in the stabilization of the structure of the matrix of keratin and gliadin films and their blends, and they are in agreement with previous works [21,22].

**Figure 2:**
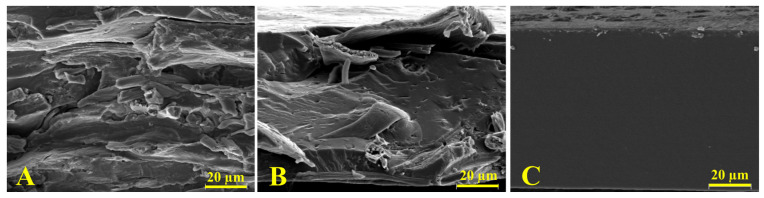
SEM micrographs of cross sections of (**A**) feathers; (**B**) the G50F50 film; and (**C**) gliadin film. Note: Magnification was 1000x.

Figure 2 shows the SEM images of cross-sectional films. Since gliadin films presented a smooth and homogeneous structure, the effect of the incorporation of feathers into the polymeric matrix films was clear. As Figure 2C shows, the gliadin film exhibited a continuous structure associated with an adequate polymeric melting process and formation of the polymeric network during the film formation through the thermocompression process. On the contrary, the film containing pure feathers (Figure 2B) evidenced a heterogeneous surface with fibrous structures, indicative of an imperfect reduction of the keratin filaments. In turn, the SEM micrograph of the film resulting from the equal mixture of gliadin and feathers presented characteristics of both polymers with both continuous and fibrous zones, indicating a heterogeneous mixture of both polymers.

### 2.2. Optical Properties

The color parameters of the blended films and their controls were analyzed, and the results are given in Table 1. The first finding was that the films’ thickness greatly increased with the feather content in the blends, presenting the F100 film more than doubling in thickness compared to the gliadin G100 film. All films were processed at the same temperature and pressure, so it is possible that the keratin in the feathers exerted greater resistance to film compression–formation. Regarding the optical parameters, all film samples were homogeneous and transparent, as the luminosity values (L*) indicated. All of the developed films displayed high values of L* close to 90, although the incorporation of feathers (and the subsequent thickness increase) slightly reduced this value, that is, slightly increasing the opacity. All of the films exhibited a visually similar yellow-greenish color that increased with the feather ratio, confirmed by increases in negative a* (green) and positive b* (yellow) values. The intensity of color depended on feathers presence; as can be seen in Table 1, the chroma value is higher in F100 than in G100 and increased in blends as the feather concentration increased.

### 2.3. Swelling, Weight Loss and Dimensional Stability Results of Developed Films

**Figure 3:**
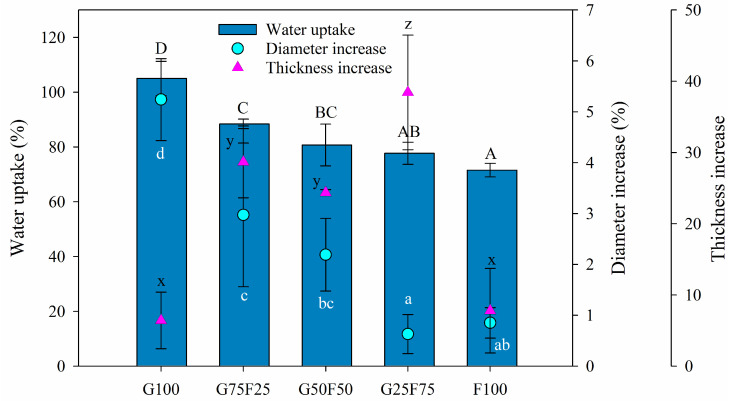
Water uptake (%), diameter increase (%), and thickness increase (%) of blended gliadin-feather films when exposed to water at 25 °C for 24 h. Labels A, B, C and D indicate statistically significant differences in water uptake results among the films; labels a, b, c and d indicate statistically significant differences among the diameter increase values between films; and labels x, y, and z indicate statistically significant differences among the values of thickness increase between films.

The swelling property provides insight into the behavior of films in aqueous medium. In this section, the swelling degree was characterized by the water uptake and the increase of thickness and diameter. As Figure 3 shows, the swelling degree of the gliadin G100 film was greater than that of feather-containing films, resulting in both the highest water uptake and the greatest diameter increase, even though the thickness was slightly increased. The G100 film approximately doubled its weight with the water uptake after 24 h. On the other hand, the feather F100 film presented the lowest water uptake and the lowest diameter and thickness increases, while the blends resulted in structures with a high swelling degree confirmed by high water uptake values and thickness increases. Blending the two matrixes resulted in intermediate water uptake and diameter increase data between both pure polymers, reducing the swelling as the proportion of feathers in the mixture increased. The thickness increase was very low for the individual polymers G100 and F100 but was high for the blends, probably due to the heterogeneous structure shown in Figure 2B.

The water resistance of the developed films in a buffer medium at pH 7 was analyzed through the weight loss (WL) parameter, the results of which are shown in Table 1. All of the samples maintained their integrity, and a slight enhancement of water resistance was observed for blended films, probably due to some crosslinking formation between some of the chemical groups of gliadin and feathers, as was previously demonstrated by the SDS-PHAGE patterns of blends. As Balaguer et al. (2011) previously observed for gliadin films, the weight loss of gliadin and feather films was also principally attributed to the release of glycerol, a hydrophilic plasticizer added on both polymers, into the aqueous medium and the loss of some polypeptide chains from gliadins and polar components from feathers [23].

### 2.4. Modulated Differential Scanning Calorimetry (MDSC) Results

**Figure 4:**
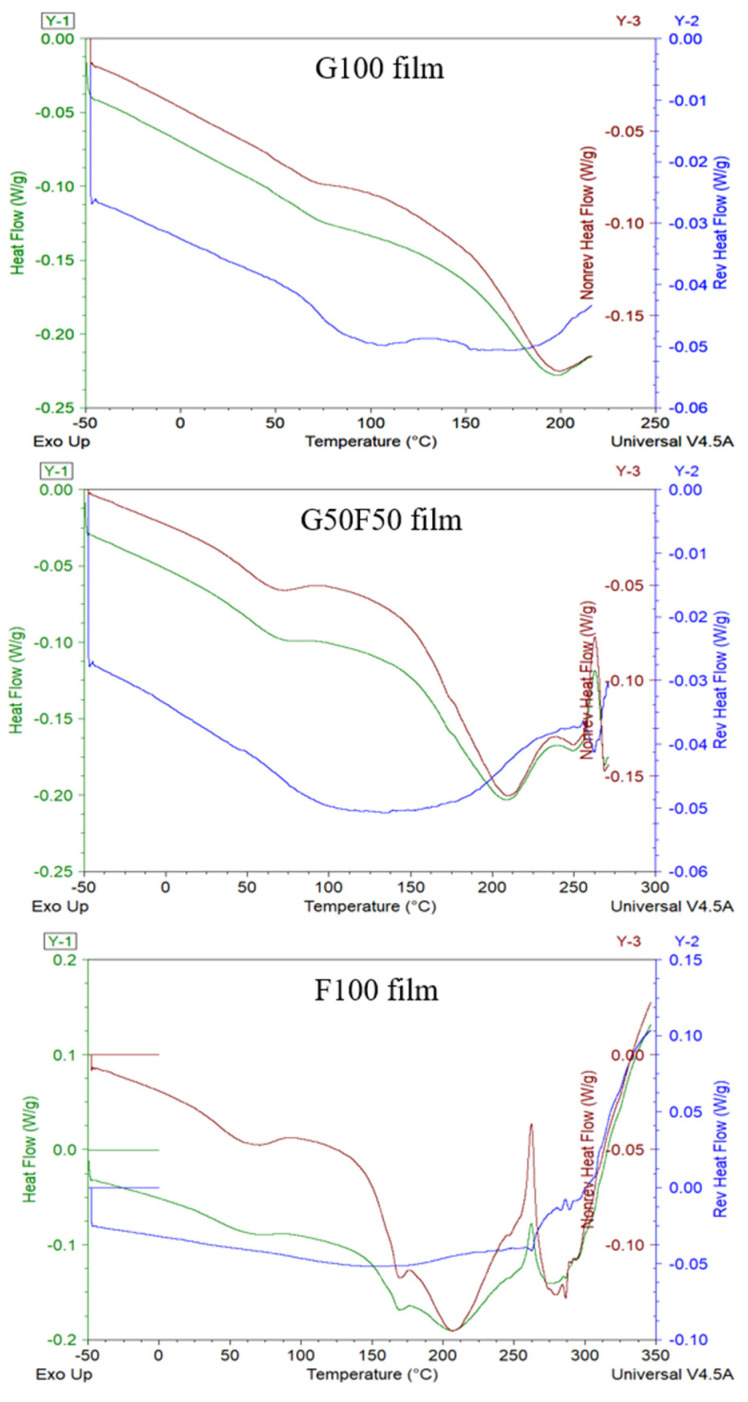
MDSC thermograms including total thermogram in green, reversible in blue lines, and non-reversible transitions in the red lines of the G100; G50F50; and F100 films.

Modulated DSC (MDSC) studies have been carried out in order to facilitate the analysis of the glass transition temperature, T_g_, of the films, since near this temperature range there are often interferences with some endothermic processes due to the intrinsic moisture evaporation and endothermic relaxation peak of internal molecular stresses occurring during processing. MDSC describes both reversible and non-reversible transitions that are correlated to the thermodynamic and kinetic contributions, respectively, in a single scan [23,24]. The MDSC conditions used in these thermograms are commonly applied for the study of glass transition temperatures with enthalpy relaxations [25]. As Figure 4 shows, the separation of the glass transition and the enthalpy relaxation was clear, and the T_g_ of G100 and G50F50 was visible as a step change on the reversing heat capacity signal while the enthalpy relaxation appeared on the non-reversing heat–flow curve.

Some authors have claimed that the molecular dynamics of vegetable proteins such as gliadins differ significantly from those of animal proteins such as keratin [26]. As Figure 4 shows, gliadin is an amorphous biopolymer that presents a clear glass transition temperature (T_g_) at ca. 73.7 °C and that evinces the transition from a glassy to a rubbery state, while feather-containing keratin does not manifest this thermal transition. This result could be due to the fact that in this work feathers are not a pure extracted polymer and the other feather components can probably hide this transition. Moreover, Ferrari and Johari (1997) have argued that the animal proteins may show exceptionally broad glass-softening endotherm only after they are hydrated, and, in contrast to gliadins, this endotherm was not affected by the amount of water. On the other hand, hydration of vegetable proteins, as the addition of plasticizers, shifts the glass transition temperature lower [27].

In Figure 4, the total heat flow of each sample in green lines is the sum of reversing and non-reversing heat flows. The non-reversible processes (in red lines) displayed the molecular relaxation and evaporation processes of both gliadin and feathers. Conversely, the reversing events (in blue lines) involved the glass transition temperatures. Analysis of the T_g_ of blends indicated that all samples presented similar values, approximately between 73.6 and 75.6 °C. MDSC did not provide relevant information, but it evidenced the lack of crystallinity of both polymers and the lack of effect of feather incorporation on the T_g_ values of gliadins in the blended films. On the other hand, feather-containing films presented a small endothermic transition in the non-reversible transition associated probably to the intrinsic water evaporation. Subsequently, a broad endothermal first-order transition due to helix denaturation protein was revealed between 150 and 250 °C with a maximum at approximately 208 °C [28,29].

### 2.5. Mechanical Properties of Developed Films

**Figure 5:**
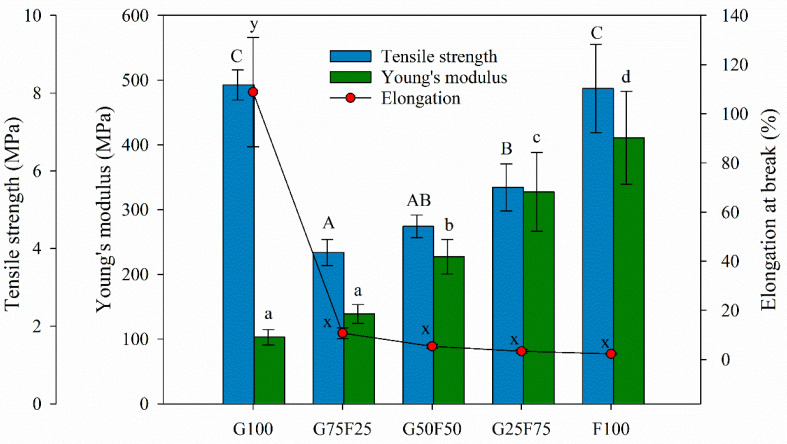
Tensile parameters of gliadin (G), feather (F) film, and their blends. Labels A, B, and C indicate statistically significant differences among the tensile strength values between films; labels a, b, c and d indicate statistically significant differences among the Yount’s modulus values between films; and labels x and y indicate statistically significant differences among the values of elongation at break between films.

The tensile parameters of gliadin and feathered films and their blends are detailed in Figure 5. The F100 film presented the highest YM value, in agreement with other works [30]. Minimizing the extraction process of keratin from feathers, the protein maintained the disulfide and hydrogen bonds. The Young’s modulus values of the blended films were between the values of the gliadin (G100) and feather (F100) control films.

Although the gliadin film also exhibited high tensile strength, when both matrices were blended, this parameter was highly affected, probably due to the lack of homogeneity and cohesion of the samples, resulting in breaking points. However, as the concentration of feathers increased, the Young’s modulus and tensile strength of blended films increased. Keratins are the chemical base of animals’ tissues and have mainly structural and mechanical functions. They present good mechanical resistance thanks to the large number of disulfide bonds that are formed by covalent links among polypeptide chains present in the protein [17]. In general, feather films presented higher stiffness than gliadins, similar maximum tensile strength at break, and lower deformation due to their keratin composition.

The incorporation of feather into the blends imparted a significant decrease on the elongation at break values of the developed films. Possibly, the heterogeneous morphology of the blended matrices generated shear points that resulted in materials with low fracture toughness. Generally, the mechanical properties of polymer blends are known to be influenced by the interaction or compatibility between the component polymers [31]. Usually, compatible polymer blends lead to a significant improvement in mechanical properties, while incompatible polymer blends often lead to inferior mechanical properties [32]. The low elongation at break value of the F100 film suggests that the addition of 20 wt%. glycerol was not sufficient to overcome the brittleness of this material. Gliadin and keratin films obtained by the casting technique have shown higher elongation at break values [30]. During processing by thermopressing, due to the high temperature, a loss of plasticizer could probably have occurred. Elongation at break values could be raised by increasing plasticizer concentration due to their ability to reduce hydrogen bonds between polymeric chains and decrease protein chain-to-chain interactions.

### 2.6. Barrier Properties of Developed Films

**Figure 6:**
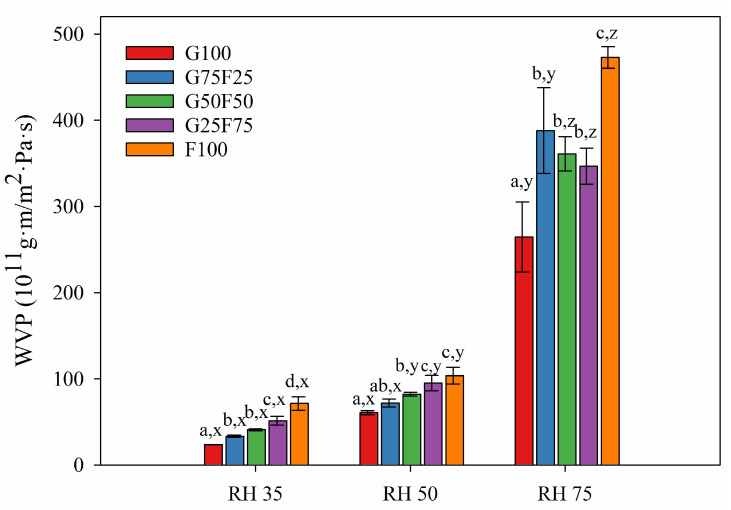
Water vapor permeability values of G and F blends at different relative humidity (RH) gradients and 23 °C. Labels a, b, c and d indicate significant differences among the values of the films at the same RH, and x, y, and z indicate significant differences among the values of WVP at different RHs of a sample.

**Figure 7:**
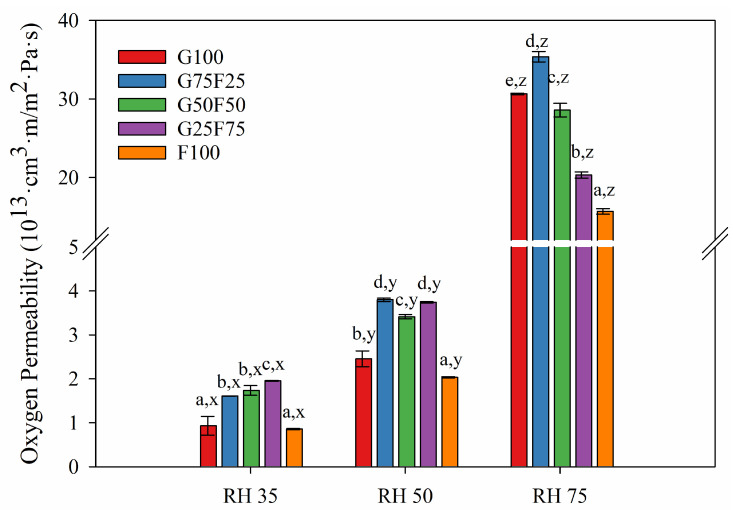
Oxygen permeability (OP) values of G and F blends at different relative humidity (RH) gradients and 23 °C. Labels a, b, c, d and e indicate significant differences among the OP values of the films at the same RH, and x, y, and z indicate significant differences among the values of the OP of a film at different RHs.

Figure 6 and Figure 7 exhibit the water and oxygen barrier properties of the developed films, respectively. Both graphs indicate that the values of water vapor and oxygen permeabilities were dependent on the ratio of gliadin and feather and on the humidity (RH) to which the film was exposed. Both permeabilities were clearly affected by increasing RH due to the hydrophilic character of both polymers. The chemical structure of both proteins together with the addition of glycerol as a plasticizer resulted in a highly moisture-sensitive polymeric matrix due to the interaction between hydrogen bonds and water molecules, thereby reducing the interchain bonding and increasing the movement of polymeric chains, and therefore, the permeability to gases and vapor [33]. However, it should be noted that the gliadins exhibited slightly lower water vapor permeability values than the feathers, and their resulting blends displayed intermediate permeability results.

As Figure 6 shows, at RH 35 and 50, the water vapor permeabilities of blended films were between the values of the gliadin (G100) and feather (F100) control films. Increasing feather concentration deteriorated the water vapor barrier of gliadin films. The hydrophilic nature of proteinaceous films and the substantial amount of hydrophilic plasticizer added to impart adequate flexibility were responsible for their poor water vapor resistance [34]. Glycerol is particularly more hydrophilic than other plasticizers such as sorbitol whose addition to keratin films has shown lower WVP values [30].

In contrast to water vapor permeability, feather fibers showed a higher oxygen barrier than gliadins. The oxygen permeability values decreased greatly with the incorporation of 75% feather at high RH. Gliadins present an excellent oxygen barrier in dry conditions, but as Figure 7 shows, this property was greatly deteriorated at RH 75. Specifically, for a gliadin polymeric matrix, the entry of water molecules and their interaction with the amide groups of the polymer progressively reduce the cohesive energy of the polymer through interchain hydrogen bonds, resulting in plasticization of the matrix and increasing the diffusion coefficient [34].

## 3. Discussion

The present study aimed to analyze the viability of developing food packaging materials based on gliadin and feather fibers. Morphological analysis revealed that the miscibility of both polymeric matrices was not completely homogenous, and therefore, several physical properties were dependent on the gliadin and feather content. Gliadins evidenced higher hydrophilicity and therefore higher water uptake and diameter increase values in aqueous medium. In general, all of the blended films presented low elongation at break values and their stiffness increased with feather content.

At low and medium relative humidities (RH), gliadin G100 film presented the lowest water vapor permeability, and as the concentration of feather increased, WVP increased. At high RH, the WVP of films did not present significant differences. In contrast, the oxygen permeability (OP) of films at low and medium RHs were similar, while at high RH, OP values diminished greatly as feather concentration increased.

## 4. Materials and Methods

### 4.1. Materials

Crude wheat gluten (>80% protein), glycerol, ethanol, tris-hydrochloride (Tris-HCl), sodium dodecyl sulfate (SDS), ethylenediaminetetraacetic acid (EDTA), bromophenol blue, 2-mercaptoethanol (2-ME), and sodium sulfite were supplied by Sigma (Madrid, Spain). Chicken feathers were purchased from the Featherfiber Corporation^®^ (Nixa, MO, USA).

### 4.2. Gliadin and Feather Keratin Resin Preparation

The gliadin-rich fraction was extracted from wheat gluten according to the method described by Hernández–Muñoz et al. (2003) [35]. Initially, 100 g of wheat gluten was dispersed in 400 mL of 70% (*v*/*v*) ethanol/water mixture, stirred overnight at room temperature, and centrifuged at 5000 rpm for 20 min at 20 °C. The supernatant containing the gliadin-rich fraction was collected, and the precipitate, consisting mostly of glutenins and residual starch, was discarded. The protein content in the supernatant was determined gravimetrically after solvent evaporation. Glycerol was added to the gliadin-rich fraction at a ratio of 25 g/100 g of protein and was stirred until complete mixing. The mixture was left to evaporate under continuous stirring at 100 °C for 15 h and was then placed in a thermostatic chamber at 37 °C for one week until complete evaporation of the solvent was achieved. The resin obtained was frozen with liquid nitrogen and ground in a Moulinex grinder to obtain a fine powder.

Crude feathers (10 g) were mixed with 6 mL of 5% (*w*/*v*) sodium sulfite aqueous solution (final concentration of 3 g Na_2_SO_3_/100 g feather) and 10 mL of distilled water, and the blend was manually stirred in a mortar for 10 min. Then, 2.5 g of glycerol (final concentration of 20 g glycerol/100 g feather) was added and consequently blended. The blend obtained was dried under vacuum at 70 °C for 24 h, stored at 0% RH for 5 days, and then ground in a Moulinex grinder (AR110830, 180 W, Madrid, Spain) to obtain a fine powder. The powders were kept under dry conditions until their use.

### 4.3. Film Formation

The powders of plasticized resins made from gliadin and feathers were blended at 4:0, 3:1, 2:2, 1:3, and 0:4 (*w*/*w*) ratios in a mortar through manual mixing for 7 min. The powder mixture was conditioned at 50% RH, and then approximately 0.9 g of each blend was thermally compressed using a hydraulic press (Carver, Inc., Wabash, IN, USA) at 130 °C. Pressure was applied first at 4 t for 1 min, subsequently at 14 t for 2 min, and finally at 22 t for 9 min. The resulting films were coded as G100 (100% gliadin), G75F25 (75% gliadin, 25% feather keratin), G50F50 (50% gliadin, 50% feather keratin), G25F75 (25% gliadin, 75% feather keratin), and F100 (100% feather keratin). The resulting films were conditioned and stored at 50% RH until their characterization.

### 4.4. Sodium Dodecyl Sulfate-Polyacrylamide Gel Electrophoresis (SDS-PAGE)

The molecular weight distribution of proteins present in films was analyzed by SDS-PAGE performed in a vertical electrophoresis unit (Bio-Rad Laboratories, Hercules, CA, USA) and following the procedure of Laemmli with some modifications [36]. Samples were denatured by mixing 50 µg of ground film with 1 mL of loading buffer (2.5% SDS, 10 mM Tris-HCl, 1 mM EDTA, 6% glycerol, 0.01% bromophenol blue, with or without 5% 2-ME) with or without the reduction of disulfide bonds with 5% (*v*/*v*) 2-mercaptoethanol and heating at 95 °C for 5 min. Each sample–buffer mixture was allowed to stand at room temperature for 2 h with occasional shaking and was centrifuged at 13,000× *g* for 10 min. A total of 10 µL of the clear top layer of each sample was loaded into each slot in the gel. The stacking gel was 4% acrylamide, and the resolving was 12% acrylamide. The electrophoresis was carried out at 25 mA/gel for 1.5 h. The gels were stained with Coomassie brilliant blue. The molecular weights of the standard protein mixture (Bio-Rad) ranged from 199 kDa (myosin), 116 (β-galactosidase), 97 (bovine serum albumin), 53 (ovalbumin), 37 (carbonic anhydrase), 29 (soybean trypsin inhibitor), 20 (lysozyme), to 7 kDa (aprotinin).

### 4.5. Characterization of Developed Films

The thickness of the films was measured using a micrometer (Mitutoyo, Neuss, Germany) with a sensitivity of ±1 µm. The mean thickness was calculated from measurements taken at ten different locations on each film sample.

#### 4.5.1. Optical Properties

Film color was measured using a Konica Minolta CM-35000d spectrophotometer set to D65 illuminant/10° observer (Minolta CO., Tokyo, Japan). Film specimens were measured on the surface of a standard white plate, and the CIELAB color space was used to obtain the color coordinates L* [black (0) to white (100)], a* [green (−) to red (+)], and b* [blue (−) to yellow (+)]. The color coordinate C* is the chroma, and it was calculated according to Equation (1):

C* = (a*^2^ + b*^2^)^1/2^ (1)

Eight measurements were taken of each sample, and three samples of each film were measured.

#### 4.5.2. Morphological Analysis

The films were fractured under liquid nitrogen, and their cross-section surface morphology was studied by scanning electron microscopy (SEM) using a Hitachi model S-4100 with a BSE Autrata detector and an EMIP 3.0 image capture system (Hitachi, Madrid, Spain). The samples were coated with gold–palladium under vacuum in a sputter coating unit.

#### 4.5.3. Swelling Property, Weight Loss and Dimensional Stability

Film specimens measuring 25 mm in diameter were dried for 10 days in a desiccator at 0% relative humidity (RH). Samples were accurately weighed (initial dry weight, $W_{d}^{i}$) and immersed in test tubes containing 20 mL of 0.1 M sodium phosphate buffer pH 7. The tubes were agitated at 180 rpm and 25 °C for 24 h. Films were removed from the solutions; the remaining water was eliminated from the surface with absorbent paper before weighing (final wet weight, $W_{w}^{f}$). Films were placed in the desiccator until they reached a constant weight (final dry weight, $W_{d}^{f}$). The percentage of weight loss of the films and water uptake were calculated according to Equations (2) and (3):

(2) $$Water\;uptake\;\%=(W_{w}^{f}-W_{d}^{f})/(\;W_{d}^{f})\cdot 100$$

(3) $$Weight\;Loss\;\%=(W_{d}^{i}-W_{d}^{f}f)/(\;W_{d}^{i})\cdot 100$$

As a measure of the dimensional stability of the films after immersion, both the diameter and the thickness were measured, and results were expressed as the diameter and thickness increase, ∆∅ and ∆l, respectively, calculated by Equations (4) and (5):

(4) $$∆\emptyset \;(\%)=(\emptyset^{f}-\emptyset^{i})/\;\emptyset^{i}\cdot 100$$

(5) $$∆l\;(\%)=(l^{f}-l^{i})/\;l^{i}\cdot 100$$

where $\emptyset^{i}$ is the initial diameter, $\emptyset^{f}$ is the final diameter, $l^{i}$ is the initial thickness, and $l^{f}$ the final thickness.

#### 4.5.4. Modulated Differential Scanning Calorimetry (MDSC)

Measurements of the glass transition temperature (T_g_) of films conditioned at 0% RH at 23 °C were determined by modulated differential scanning calorimetry using a TA Instruments DSC Q2000 (TA Instruments Inc., New Castle, DE, USA) equipped with Universal Analysis 2000 software. Nitrogen was used as a purge gas at a flow rate of 50 mL/min. Temperature calibration of the instrument was performed with indium. The heating rate was 2 °C/min, the modulation period was 60 s, and the amplitude of modulation was 0.32 °C. The glass transition temperature was recorded from the inflexion point of the reversing heat flow signal. The films were dried over P_2_O_5_ at 23 °C for 2 weeks before testing. Dry samples of approximately 5 mg were placed in aluminum pans with inverted lids in order to achieve optimum thermal conductivity. These were sealed, punctured three times, and kept over P_2_O_5_ for a further week prior to scanning. All samples were measured in duplicate.

#### 4.5.5. Mechanical Properties

The mechanical behavior of the developed films, such as the Young’s modulus (YM), tensile strength (TS), and elongation at break (EB), were determined in a BDO-FB 0.5 TH universal machine (Zwick Roell, Ulm, Germany) according to the ASTM D882 normative [37]. Specimens with dimensions 16.5 cm × 2.4 cm were cut and conditioned at 23 °C for 48 h in a desiccator at 50% relative humidity. The separation distance between the jaws was 50 mm, and the crosshead speed was 500 mm/min. The results were reported as the mean and standard deviation of 15 measurements.

#### 4.5.6. Water Vapor Permeability

The water vapor transmission rates (g/m^2^·s) of developed films were measured using a PERMATRAN-W Model 3/33 (Lippke, Neuwied, Germany). Testing was performed at 23 °C with an RH gradient of 50 to 0% (in dry nitrogen) across the film. At least four samples of each type of film were analyzed. Permeability values were reported as the water permeability coefficient in g·m/m^2^·s·Pa calculated using Equation (6):

P = (Q·l)/(A·t·∆P)(6)

Q is the amount of permeant passing through a film (g) of thickness l (m) and area A (m^2^); t is time (s), and ∆P is the partial pressure differential across the film (Pa). ∆P is calculated from the water vapor partial pressure at the temperature selected and the RH gradients.

#### 4.5.7. Oxygen Permeability

Oxygen transmission rates (cm^3^/m^2^·s) through films were measured using an OX-TRAN Model 2/21 (Lippke, Neuwied, Germany) according to the ASTM Standard Method D 3985-05 [38]. Testing was performed at 23 °C and constant relative humidities of 35%, 50%, and 75%. At least four samples of each type of film produced were analyzed. The samples were conditioned in the cells for 24–48 h, then the transmission values were determined every 45 min. Permeability values were reported as the oxygen permeability coefficient in cm^3^·m/m^2^·s·Pa.

### 4.6. Statistical Analysis

Statistical analysis of the results was performed with Statgraphics Plus 5.1. commercial software (SPSS Inc., Chicago, IL, USA). A one-way analysis of variance (ANOVA) was carried out. Differences between means were assessed on the basis of confidence intervals using the Tukey test at a level of significance of *p* ≤ 0.05. The data were graphically plotted with SigmaPlot 14.5 software (Systat Software Inc., Richmond, CA, USA).

## 5. Conclusions

Blended films composed of gliadin and chicken feather fibers were successfully developed at different ratios. The findings of this work indicate potential for the application of the developed blends in food packaging systems, although further analysis is needed. The hydrophilic nature of both polymeric matrices resulted in the blended films having a significant swelling degree. Analysis of the mechanical and barrier properties revealed that these properties can be modified by adjusting the concentration of both polymeric matrices. Tensile tests indicated poor miscibility between the polymeric proteins, confirming the findings observed through the scanning electron microscopy analysis. Although the addition of the feathers increased the stiffness of the material, as confirmed by an increase in Young’s modulus, it implied a loss of elasticity of the blends. Given that these materials were obtained through the application of high temperatures that could imply a loss of plasticizer during processing, the low elongation at break values of the blended films could be raised by increasing the plasticizer concentration. The oxygen permeability results indicated the suitability of the fabricated blends for gas barriers that could be part of a multilayer system, indicating potential application in novelty and ecofriendly packaging. Additionally, as a positive relevant point, blending chicken feathers directly with biopolymers could be an alternative method of upcycling this waste by-product.

## Figures and Tables

**Table 1:** Thicknesses, color parameters and weight losses (WL) of gliadins and feather blend materials.

Sample	Thick (µm)	L*	a*	b*	C*	WL (%)
G100	76.8 ± 6.3 ^a^	89.9 ± 0.3 ^c^	−2.29 ± 0.05 ^d^	9.60 ± 0.64 ^a^	9.9 ± 0.6 ^a^	26.1 ± 0.6 ^c^
G75F25	86.3 ± 7.3 ^a^	89.7 ± 0.3 ^cd^	−2.28 ± 0.03 ^d^	10.35 ± 0.78 ^ab^	10.6 ± 0.8 ^ab^	26.5 ± 0.1 ^c^
G50F50	92.5 ± 11.3 ^a^	89.2 ± 0.3 ^c^	−2.46 ± 0.06 ^c^	11.35 ± 0.94 ^b^	11.6 ± 0.9 ^b^	25.9 ± 1.3 ^bc^
G25F75	123.1 ± 9.1 ^b^	87.6 ± 0.3 ^b^	−2.71 ± 0.05 ^b^	13.94 ± 1.10 ^c^	14.2 ± 1.1 ^c^	23.7 ± 1.4 ^a^
F100	180.4 ± 12.5 ^c^	86.4 ± 0.4 ^a^	−2.95 ± 0.03 ^a^	15.75 ± 0.83 ^d^	16.0 ± 0.8 ^d^	24.1 ± 1.0 ^ab^

Labels a, b, c and d indicate statistically significant differences among the values of the same property in the same row.

## Data Availability

Not applicable.
